# Combined modality therapy of central nervous system tumours

**DOI:** 10.1054/bjoc.2001.2134

**Published:** 2001-11

**Authors:** R Rampling

**Affiliations:** Neuro-Oncology, Beatson Oncology Centre, Glasgow, UK


*British Journal of Cancer* (2001) **85**(11), 1809–1810
© 2001 Cancer Research Campaign
doi: 10.1054/ bjoc.2001.2134, available online at http://www.idealibrary.com on IDEAL

Review

### Combined modality therapy of central nervous system tumours

**Petrovich et al**

Springer Verlag: Berlin, 2001 pp 226 ISBN 3540 660534 Price: £170.00

In reviewing any technical book it is inevitable to approach it with some preconceptions. The title ‘Combined Modality Therapy of Central Nervous System Tumours’ would lead me to expect some introductory chapters that describe the tumours themselves with an introduction to the techniques involved in their management. I would expect the bulk of the book to comprise a critical review of current management techniques for individual tumours with a view of how these may be integrated into a state of the art, multi-modality approach to therapy. Such a book involving many authors needs careful editing if it is to make sense. How does this book match up?

The introductory chapters are much as expected but are of mixed quality. An excellent chapter on epidemiology is followed by an inadequate, incomplete and poorly referenced pathology chapter. There are three very good chapters on radiobiology, the physics of external beam radiotherapy and stereotaxy that introduce basic principles in radiotherapy. The chapter on stereotaxy is particularly good. This introduction works very well and facilitates an understanding of the radiotherapy applications that arise in later chapters. Strangely, however, there is no similar introduction to surgical or chemotherapy techniques and the reader is left to fend for himself when some quite complex ideas with respect to these modalities occur in later, tumour orientated chapters. Likewise, the imaging chapter represents a missed opportunity. There is no useful introduction to the standard techniques and no worthwhile discussion of the newer imaging methods (functional MRI, PET, and SPECT) and how they may be incorporated into the management of brain tumours. Rather, the chapter takes a familiar approach of describing many different tumour types in terms of MRI based imaging (T1, T2 and T1/Gd appearances) and includes a lot of unnecessary clinical material which is available elsewhere in the book. Also surprising is the failure to discuss certain rare tumours that have characteristic imaging fingerprints such as central neurocytoma and gliomatosis cerebri.

There follows a series of chapters dedicated to the management of individual tumours. These are wide ranging and cover benign tumours (pituitary tumours, craniopharyngioma, vestibular Schwannoma and meningioma), the more common malignant tumours and rarer tumours, though many of these latter only briefly. Some of the common tumours are given separate chapters devoted to surgical, radiotherapeutic and, sometimes, chemotherapeutic perspectives. This format does produce some very good authoritative writing in places but inevitably leads to considerable and annoying repetition. For example Simpson grading for meningioma is listed twice in consecutive chapters and the principles of stereotactic radiotherapy appear ubiquitously! The balance of these chapters is uneven, as if the authors had not read their editor’s instructions. In some we are given a great deal of technical detail on how a particular procedure is performed, in others very little. Some convincing chapters are spoiled when the author succumbs to the temptation to wander away from his own discipline with accompanying loss of authority. For example, the surgeons writing on pituitary treatment attempt to discuss radiotherapeutic management using outdated references and end by giving advice that is immediately contradicted in the following (radiotherapy) chapter.

There are some gems to be found. Chapter 14, on low grade brain tumours, tackles a very difficult subject extremely well. It is thoughtful and comprehensive in its literature review. It discusses the value of individual therapies without contradiction and offers guidelines that are clear, workable and credible. It is a model of how to write a chapter on multi-modality therapy. Unfortunately, not all chapters address the task in the same way, nor are they so objective. For example Chapter 23, on primary CNS lymphoma, spends more time discussing presentation than treatment and fails to give any clear guidelines on management.

There are many additional annoyances in this book. Although it is comprehensively referenced, many of the articles are described in the body of the text which makes for difficult and tedious reading. Whilst this can be justified if adequate criticism of the referenced article is given, often this is not the case and the information could have been better delivered in the form of carefully constructed tables. There are too many examples of ‘loose’ writing. Stereotactic irradiation is not the ‘delivery of a single high doses of radiation’; rather it is a means of irradiation which uses stereotactic localization techniques. Personal prejudice too often overrides objectivity. It is irresponsible to say that ‘clinical studies have shown a consistent improvement in two year survival (*in glioma*) with the use of a stereotactic boost.’ when no appropriate randomized studies have been done and selection bias could equally well explain the results. The allocation of space is uneven. Craniopharyngioma is given 2 chapters, 42 pages and 5 authors, whilst pineal region tumours, which demand high levels of multi-modality integration in their management, are distributed in small sections over several chapters (on paediatrics, rare tumours and chemotherapy) without any attempt at integration.

Whilst there is much in this book that is good, the format and poor editing lead, on the one hand, to much repetition and on the other to the omission or under-representation of some important topics. There is conflicting advice, sometimes in adjacent chapters, which may be very confusing to the newcomer to neuro-oncology. There are already good books available on many of the topics covered here and in spite of its title, this one does not satisfy the intended niche. In the end this is probably more a book for the already experienced practitioner who can read through the bias of some of the individual contributors to get at some of the very good information that is available. Future editions should concentrate on smoothing out the writing and providing a true multi-modality perspective.

**R Rampling**
*Professor of Neuro-Oncology*
*Beatson Oncology Centre*
*Glasgow, UK*

*© 2001 Cancer Research Campaign*

